# Development of Machine Learning Algorithms for Identifying Patients With Limited Health Literacy

**DOI:** 10.1111/jep.14248

**Published:** 2024-11-22

**Authors:** Dylan Koole, Oscar Shen, Amanda Lans, Tom M. de Groot, J. J. Verlaan, J. H. Schwab

**Affiliations:** ^1^ Department of Orthopaedic Surgery, Orthopaedic Oncology Service Massachusetts General Hospital, Harvard Medical School Boston Massachusetts USA; ^2^ Department of Orthopaedic Surgery, Leiden University Medical Center Leiden University Leiden The Netherlands; ^3^ Department of Orthopaedic Surgery, University Medical Center Utrecht Utrecht University Utrecht The Netherlands; ^4^ Department of Orthopaedic Surgery, University Medical Center Groningen University of Groningen Groningen The Netherlands

**Keywords:** health literacy, machine learning, orthopaedic surgery, social determinants of health, spine

## Abstract

**Rationale:**

Limited health literacy (HL) leads to poor health outcomes, psychological stress, and misutilization of medical resources. Although interventions aimed at improving HL may be effective, identifying patients at risk of limited HL in the clinical workflow is challenging. With machine learning (ML) algorithms based on readily available data, healthcare professionals would be enabled to incorporate HL screening without the need for administering in‐person HL screening tools.

**Aims and Objectives:**

Develop ML algorithms to identify patients at risk for limited HL in spine patients.

**Methods:**

Between December 2021 and February 2023, consecutive English‐speaking patients over the age of 18 and new to an urban academic outpatient spine clinic were approached for participation in a cross‐sectional survey study. HL was assessed using the Newest Vital Sign and the scores were divided into limited (0–3) and adequate (4–6) HL. Additional patient characteristics were extracted through a sociodemographic survey and electronic health records. Subsequently, feature selection was performed by random forest algorithms with recursive feature selection and five ML models (stochastic gradient boosting, random forest, Bayes point machine, elastic‐net penalized logistic regression, support vector machine) were developed to predict limited HL.

**Results:**

Seven hundred and fifty‐three patients were included for model development, of whom 259 (34.4%) had limited HL. Variables identified for predicting limited HL were age, Area Deprivation Index‐national, Social Vulnerability Index, insurance category, Body Mass Index, race, college education, and employment status. The Elastic‐Net Penalized Logistic Regression algorithm achieved the best performance with a c‐statistic of 0.766, calibration slope/intercept of 1.044/−0.037, and Brier score of 0.179.

**Conclusion:**

Elastic‐Net Penalized Logistic Regression had the best performance when compared with other ML algorithms with a c‐statistic of 0.766, calibration slope/intercept of 1.044/−0.037, and a Brier score of 0.179. Over one‐third of patients presenting to an outpatient spine center were found to have limited HL. While this algorithm is far from being used in clinical practice, ML algorithms offer a potential opportunity for identifying patients at risk for limited HL without administering in‐person HL assessments. This could possibly enable screening and early intervention to mitigate the potential negative consequences of limited HL without taxing the existing clinical workflow.

## 1 Introduction

Health literacy (HL) is defined as a person's capacity to acquire, interpret, and apply information related to their medical conditions, general health, treatments, and long‐term care [1]. A previous study by our study group shows that up to 36% of orthopaedic spine patients are considered to have limited HL [2]. Limited HL can lead to poor health outcomes, such as increased hospitalization and higher mortality, psychological stress, and misutilization of medical resources [3, 4, 5, 6, 7, 8]. More specifically, orthopaedic spine patients with limited HL have worse baseline patient‐reported outcome measurement scores and lower health‐related quality of life [9, 10].

Interventions aimed at improving HL are moderately effective, especially in socioeconomically disadvantaged groups [11, 12]. However, identifying patients at risk of limited HL in the clinical workflow is challenging since it requires administering HL screening tools. While it is possible to incorporate routine HL screening into the clinical workflow, issues such as low compliance and high variability lead to less reliable results [13, 14]. Moreover, healthcare professionals may experience additional strain in administering these questionnaires given their existing workload.

Before and during a patient's consultation with a physician, a substantial amount of information is already being documented. This includes basic personal information, such as age, height, and weight, as well as a patient's address and insurance plan. This data can be employed more efficiently to predict a patient's HL status when compared with more traditional HL screening tools since this data does not require additional effort or resources to be collected. While machine learning (ML) algorithms are becoming increasingly accessible, we sought to explore the potential of these algorithms in predicting which patients are more likely to possess limited HL with data that is already available or easy to obtain. In this way, healthcare professionals would be enabled to incorporate HL screening methods into the clinical workflow, without the need for administering lengthy and time‐consuming questionnaires. To our knowledge, no ML algorithm to screen for limited HL exists to date. Therefore, this study aims to develop a ML prediction model to identify patients with limited HL, exploring the potential of ML in this field and to serve as a first proof‐of‐concept for further algorithm development.

## 2 Methods

### 2.1 Guidelines

Transparent Reporting of a multivariable prediction model for Individual Prognosis Or Diagnosis (TRIPOD) and Guidelines for Developing and Reporting Machine Learning Models in Biomedical Research were followed [15, 16].

### 2.2 Data Source

**Figure 1:**
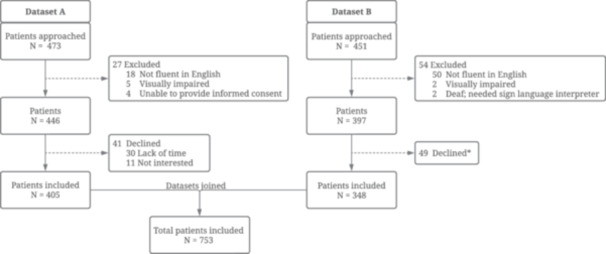
Flowchart of inclusion‐ and exclusion criteria in the combined datasets A and B. *Reason not further specified.

Two datasets from other studies by our research group were combined to have sufficient data to train ML algorithms (Figure 1). Both datasets contain prospectively collected patient data which was gathered under the approval of our institutional review board between December 2021 and March 2022 (Dataset A) and October 2022 and February 2023 (Dataset B) at a tertiary urban academic medical centre in the United States. The data was acquired by verbally administering surveys and through patients' electronic health records (Epic).

### 2.3 Study Population

The inclusion and exclusion criteria were identical in both studies. Patients over 18 years of age and new to the outpatient spine clinic were approached by trained research study staff for inclusion in the study during their first visit to the clinic, either before or after the consultation with their physician. Patients were excluded if they were visually impaired, did not consider themselves fluent in English, needed a sign language interpreter, were unable to provide informed consent, or declined participation. Verbal informed consent was obtained before any study procedures.

### 2.4 Outcome Measures and Explanatory Variables

Consenting patients were asked to verbally complete a sociodemographic survey and the Newest Vital Sign (NVS) HL assessment in both studies. HL was the primary outcome as defined by the NVS, a validated tool to assess both HL and numeracy [17, 18]. It consists of a nutritional label accompanied by six questions (Supporting Information S1: Appendix I). A score of 0–1 indicates a high likelihood (50% or more) of limited HL, a score of 2–3 indicates a possibility of limited HL, and a score of 4–6 almost always indicates adequate HL. These scores were reduced to two categories to optimize sensitivity and specificity: limited HL (score 0–3) and adequate HL (score 4–6) [18]. This categorization has been employed in other orthopaedic populations, including hand and spine patients [19, 20].

Variables collected in the sociodemographic survey in both studies were ethnicity, race, employment status, educational attainment, annual household income, current marital status, housing status, and housing insecurity. Other variables were collected through manual chart review: sex, age, Body Mass Index (BMI), insurance status, smoking status, and alcohol use at the date of enrollment. Housing insecurity was defined as: ‘Being worried or concerned about not having a place to live in the past 6 months’. Alcohol use was categorized as alcohol risk: > 2 alcoholic drink equivalents in a day for men or > 1 in a day for women [21]. Additionally, the Social Vulnerability Index (SVI) and Area Deprivation Index (ADI) were extracted using online mapping tools based on individual patient addresses [22, 23]. The ADI is based on census variables that reflect socioeconomic status and is represented as national percentile and state decile [22]. The SVI, developed by the Centers for Disease Control and Prevention, utilizes census variables to help identify communities that may need support before, during, or after disasters. SVI scores can be categorized into four categories: low (< 0.25), low‐medium (≥ 0.25–< 0.50), medium‐high (≥ 0.50–< 0.75), and high (≥ 0.75) [23].

### 2.5 Predictors

The following variables were selected as potential predictors for training algorithms based on their accessibility in patient charts or ease of being obtained during normal physician‐patient interaction: age (continuous), sex (male/female), ADI national percentile (continuous), SVI (low/low‐medium/medium‐high/high), insurance category (public/private), smoking status (active/former/nonsmoker) alcohol risk (yes/no), BMI (continuous), race (White/non‐White), ethnicity (Hispanic or Latino/not), college education (yes/no), employment (employed/not employed), and marital status (married/not married). HL category was the dependent variable in this investigation.

### 2.6 Missing Data

Rates of missing data for variables were as follows: BMI = 19 (2.5%), insurance category = 4 (0.5%), college education = 1 (0.1%), smoking status = 68 (9.0%), and alcohol consumption = 226 (30.0%). Multiple imputation was performed with the missForest package in Python software to impute variables with less than 30% missing data. This method is a nonparametric imputation method that utilizes a random forest algorithm to estimate missing values based on observed values in the data set [24]. Alcohol consumption was removed as a potential predictor due to the amount of missing data.

### 2.7 Statistical Analysis

Descriptive statistics were used to summarize the data and to compare the patient characteristics of patients with limited HL and adequate HL (Table 1). Shapiro–Wilks tests were used to check for normality. Since the only continuous variable ‘Age in years’ did not follow a normal distribution, it was reported using median and interquartile range (IQR). Categorical variables were reported as frequencies and percentages. A Mann–Whitney *U* test was performed on the continuous variable to evaluate the difference between groups. For categorical variables, *χ* ^2^ tests were conducted. A *p* value of < 0.05 was considered statistically significant.

**Table 1:** Patient characteristics stratified by HL level.

		Health literacy		
	Total	Limited (NVS ≤ 3)	Adequate (NVS > 3)	
	753 (100%)	259 (34.4%)	494 (65.6%)	*p* value
Age in years, median (IQR)	57 (42–69)	65 (51–74)	53 (38–65)	**< 0.001**
Sex				0.525
Male	358 (47.5)	119 (45.9)	239 (48.3)	
Female	395 (52.5)	140 (54.1)	255 (51.6)	
BMI category^a^				0.916
Underweight (< 18.5)	10 (1.3)	4 (1.5)	6 (1.2)	
Healthy weight (≥ 18.5–< 25.0)	236 (31.3)	81 (31.3)	155 (31.4)	
Overweight (≥ 25.0–< 30.0)	296 (39.3)	100 (38.6)	196 (39.7)	
Obese (≥ 30.0)	211 (28.0)	74 (28.6)	137 (27.7)	
Race				**< 0.001**
White	630 (83.7)	194 (74.9)	436 (88.3)	
Black or African American	36 (4.8)	28 (10.8)	8 (1.6)	
Hispanic or Latino	23 (3.1)	15 (5.8)	8 (1.6)	
Asian	46 (6.1)	14 (5.4)	32 (6.5)	
Other	18 (2.4)	8 (3.1)	10 (2.0)	
Ethnicity				**< 0.001**
Not Hispanic or Latino	721 (95.8)	239 (92.3)	482 (97.6)	
Hispanic or Latino	32 (4.2)	20 (7.7)	12 (2.4)	
Area Deprivation Index, median (IQR)				
State decile	3 (1–6)	4 (2–6)	3 (1–5)	**< 0.001**
National percentile	14 (7–24)	18 (9–26)	12 (6–23)	**< 0.001**
SVI category				**< 0.001**
Low (< 0.25)	322 (42.8)	94 (36.3)	228 (46.2)	
Low‐medium (≥ 0.25–< 0.50)	208 (27.6)	60 (23.2)	148 (30.0)	
Medium‐high (≥ 0.50–< 0.75)	131 (17.4)	53 (20.5)	78 (15.8)	
High (≥ 0.75)	92 (12.2)	52 (20.1)	40 (8.1)	
Insurance status^a^, ^b^				**< 0.001**
Public	319 (42.4)	167 (64.5)	152 (30.8)	
Private	434 (57.6)	92 (35.5)	342 (69.2)	
Education^a^				**< 0.001**
College education or more	562 (74.6)	147 (56.8)	415 (84.0)	
No college education	191 (25.4)	112 (43.2)	79 (16.0)	
Employed versus not employed^c^				**< 0.001**
Employed	448 (59.5)	101 (39.0)	347 (70.2)	
Not employed	305 (40.5)	158 (61.0)	147 (29.8)	
Married versus not married				**< 0.001**
Married or with partner	428 (56.8)	115 (44.4)	313 (63.4)	
Not married or with partner	325 (43.2)	144 (55.6)	181 (36.6)	
Smoking status^a^				**0.003**
Never smoked	460 (61.1)	140 (54.1)	320 (64.8)	
Former smoker	223 (29.6)	84 (32.4)	139 (28.1)	
Active smoker	70 (9.3)	35 (13.5)	35 (7.1)	
Alcohol risk				**0.030**
Yes	194 (25.8)	56 (21.6)	138 (27.9)	
No	333 (44.2)	127 (49.0)	206 (41.7)	
*Missing*	*226 (30.0)*	*76 (29.3)*	*150 (30.4)*	

*Note: n* (%) unless stated otherwise. Boldface type indicates statistical significance (*p* < 0.05).

^a^ Imputed missing values: BMI (*n* = 19), insurance (*n* = 4), education (*n* = 1), smoking status (*n* = 68).

^b^ Public insurance: Medicaid, Medicare, or MassHealth; Private insurance: any other health insurance.

^c^ Employed includes being (self‐)employed or student; Not employed includes retired, unemployed, or unable to work/disabled.

### 2.8 Model Development

The available data were divided into a training and testing set with a stratified 70:30 split. Feature selection was performed by random forest algorithms with recursive feature selection [25]. Five supervised ML algorithms (stochastic gradient boosting, random forest, Bayes point machine, elastic‐net penalized logistic regression, support vector machine) were developed with the subset of variables in the training set [26, 27, 28, 29]. Model performance was assessed on the training set by bootstrapping algorithm creation 1000 times and taking the means and 95% confidence intervals of the performance metrics. Model performance was also assessed in the independent testing set, which was not used for algorithm development. The metrics used for algorithm assessment were discrimination (c‐statistic), calibration (calibration slope and intercept), and overall performance (Brier score).

Discrimination assesses the model's capacity to distinguish between patients with inadequate and adequate HL and was evaluated graphically using the receiver operating curve and numerically using the c‐statistic, also known as the area under the receiver operating curve (AUC) for binary classification [30, 31, 32, 33]. Models that achieve perfect discrimination have a c‐statistic of 1, while models with performance no better than chance have a c‐statistic of 0.5.

Calibration refers to how well the predicted probabilities of the model concur with the observed probabilities in the study population and was assessed graphically with calibration plots and numerically with calibration slope and intercept [32, 33]. The calibration slope quantifies the difference between predictor effects for each model. A calibration slope larger than 1 indicates that predictions are overly extreme, overpredicting high‐risk patients and underestimating low‐risk patients. The calibration intercept assesses whether the model has a tendency to overestimate or underestimate the probability of the outcome. A positive calibration intercept indicates general overestimation of predictions by the model. Perfect models have a calibration intercept of 0 and a calibration slope of 1.

Overall model performance was assessed with the Brier score, the mean squared error between predicted probabilities and observed values [33, 34]. It combines discrimination and calibration metrics to serve as a benchmark for overall model performance [33]. The null model Brier score assigns a predicted probability for all patients equal to the prevalence of limited HL. This establishes a baseline for comparison, enabling the assessment of the predictive accuracy of the model. Brier scores closer to zero have a lower error between predictions and observed values, indicating better model performance.

Decision curve analysis was performed to evaluate the clinical utility of the model by plotting the net benefit of using the model to make clinical decisions against the net benefit of assuming that all patients have limited HL [35, 36]. This method is useful for examining the utility of prediction models and balances the potential benefit of true positive predictions against the potential harm of false positive and false negative predictions over a range of probability thresholds.

All statistical analyses were performed using Python version 3.9.12 (Python Software Foundation) programming language.

## 3 Results

Nine hundred and twenty‐four patients were approached for inclusion in this study, of which 81 patients (8.8%) were excluded because they were not fluent in English, were visually impaired, or needed a sign language interpreter. Of the remaining 843 patients, 90 (10.7%) declined participation due to lack of time or no interest (Figure 1), resulting in a response rate of 89%. In total, 753 patients were included for model development, of whom 259 (34.4%) had limited HL. In univariate analysis, patients with limited HL were more likely to be older, Hispanic/Black, have a higher BMI, live in an area with higher deprivation, have public health insurance, have less than college education, and be unemployed (Table 1).

Random forest algorithms identified age, ADI‐national, SVI, insurance category, BMI, race, college education, and employment status as predictive factors for limited HL. C‐statistics of all models were largely similar in cross‐validation of the training set (*n* = 527) and ranged from 0.768 for Support Vector Machine to 0.839 for Stochastic Gradient Boosting (Table 2). The best‐performing model for predicting limited HL based on discrimination alone was Stochastic Gradient Boosting with a c‐statistic of 0.839. Calibration slopes ranged from 0.669 for Bayes Point Machine to 1.682 for Random Forest and calibration intercepts ranged from –0.451 for Random Forest to 0.251 for Bayes Point Machine. Brier scores ranged from 0.160 for Stochastic Gradient Boosting to 0.188 for Bayes Point Machine. In comparison, the null model Brier score was 0.226.

**Table 2:** Algorithm performance in on cross‐validation of training set (95% confidence interval), *n* = 527.

Metric	Stochastic gradient boosting	Random forest	Support vector machine	Bayes point machine	Elastic‐net penalized logistic regression
AUC	0.84 (0.83, 0.85)	0.82 (0.80, 0.84)	0.77 (0.74, 0.79)	0.78 (0.76, 0.80)	0.78 (0.75, 0.80)
Calibration intercept	−0.39 (−0.44, −0.34)	−0.45 (−0.54, −0.32)	−0.01 (−0.08, 0.10)	0.25 (0.21, 0.30)	−0.06 (−0.10, 0.00)
Calibration slope	1.59 (1.51, 1.66)	1.68 (1.49, 1.81)	0.99 (0.74, 1.14)	0.67 (0.59, 0.75)	1.08 (1.00, 1.14)
Brier score	0.16 (0.16, 0.17)	0.17 (0.16, 0.18)	0.19 (0.18, 0.20)	0.19 (0.18, 0.20)	0.17 (0.16, 0.18)

*Note:* Null model Brier score = 0.23.

Abbreviation: AUC, area under the receiver operating curve.

Model performance in the independent testing set (*n* = 226) resulted in c‐statistics ranging from 0.744 for Support Vector Machine to 0.788 for Bayes Point Machine (Table 3). Calibration slopes ranged from 0.647 for Bayes Point Machine to 1.389 for Random Forest and calibration intercepts ranged from –0.260 for Random Forest to 0.264 for Bayes Point Machine. Brier scores ranged from 0.179 for Elastic‐Net Penalized Logistic Regression to 0.188 for Bayes Point Machine. The null Brier score for the independent testing set was 0.226 as well.

**Table 3:** Algorithm performance in independent testing set (95% confidence interval), *n* = 226.

Metric	Stochastic gradient boosting	Random forest	Support vector machine	Bayes point machine	Elastic‐net penalized logistic regression
AUC	0.76 (0.69, 0.79)	0.77 (0.71, 0.82)	0.74 (0.69, 0.80)	0.79 (0.74, 0.84)	0.77 (0.71, 0.82)
Calibration intercept	−0.24 (−0.38, −0.04)	−0.26 (−0.60, 0.20)	0.03 (−0.23, 0.30)	0.26 (0.18, 0.37)	−0.04 (−0.22, 0.15)
Calibration slope	1.32 (1.04, 1.54)	1.39 (0.70, 1.89)	0.93 (0.45, 1.32)	0.65 (0.46, 0.82)	1.04 (0.76, 1.31)
Brier score	0.18 (0.18, 0.19)	0.19 (0.17, 0.20)	0.19 (0.18, 0.21)	0.19 (0.17, 0.21)	0.18 (0.16, 0.20)

*Note:* Null model Brier score = 0.23.

Abbreviation: AUC, area under the receiver operating curve.

**Figure 2:**
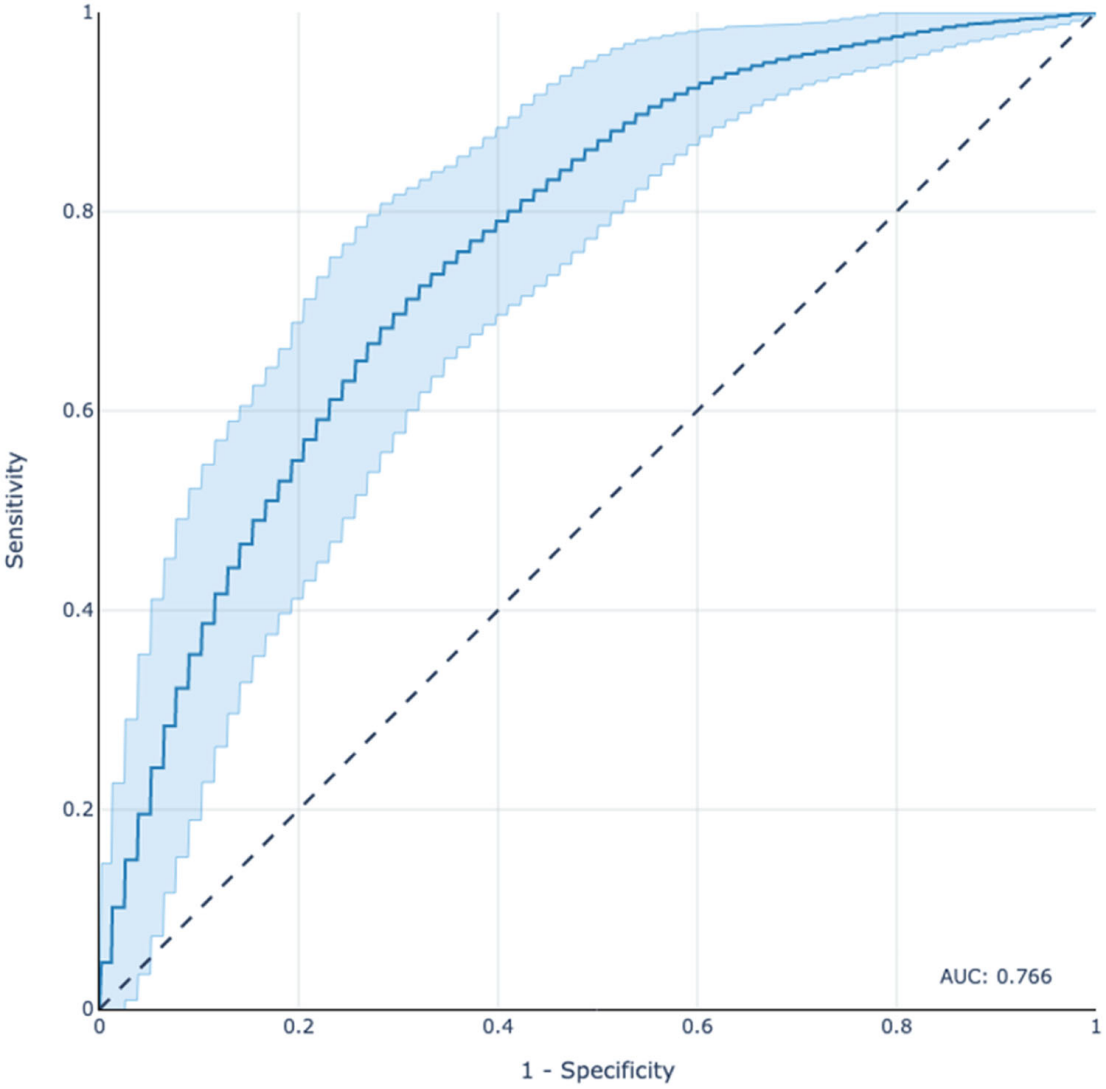
Receiver operating curve of elastic‐net penalized logistic regression model for predicting limited health literacy; testing set, *n* = 226. AUC, area under the curve.

**Figure 3:**
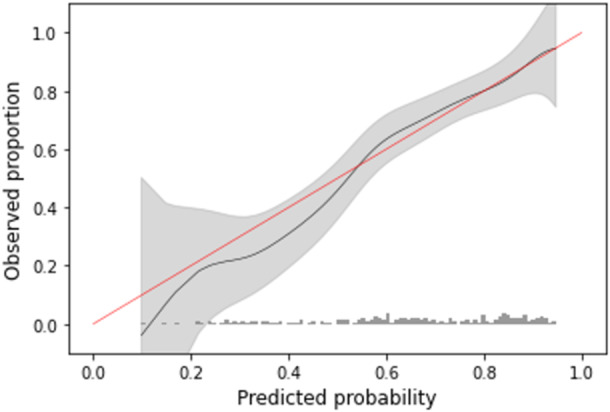
Calibration curve of elastic‐net penalized logistic regression model for predicting limited health literacy; testing set, *n* = 226.

**Figure 4:**
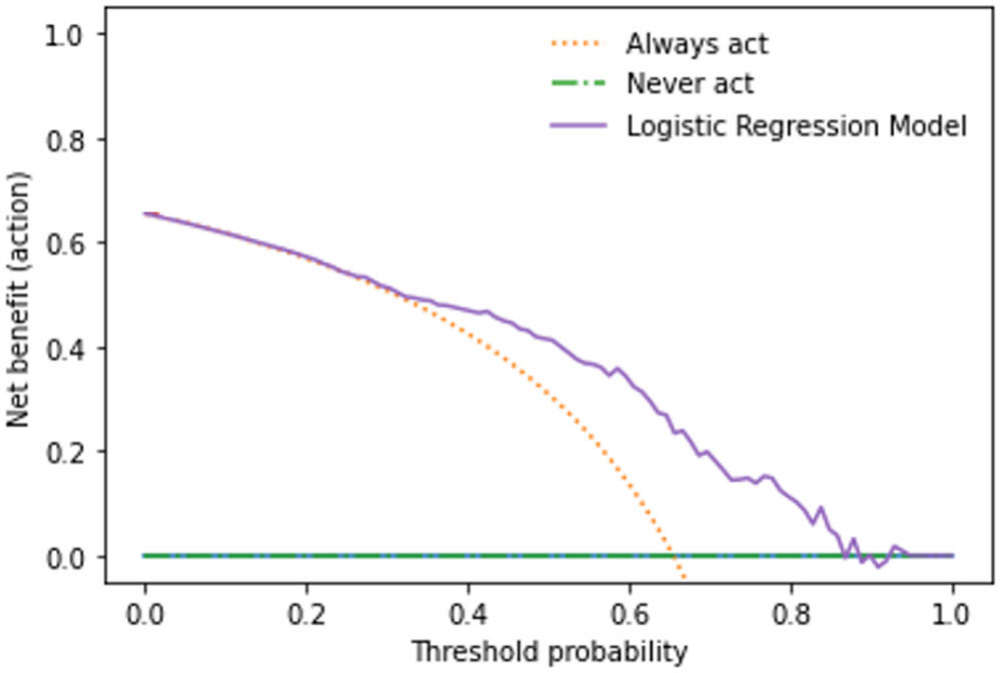
Decision analysis curve for changing management for all patients, no patients, and based on the elastic‐net penalized logistic regression model across threshold probabilities.

Elastic‐Net Penalized Logistic Regression was chosen as the final model with superior performance on calibration and overall assessment. Upon evaluation in the testing set, the model had a c‐statistic of 0.766 (Figure 2), calibration slope of 1.044 and calibration intercept of −0.037 (Figure 3), and Brier Score of 0.179. Decision curve analysis for the Elastic‐Net Penalized Logistic Regression model revealed that changing management based on the model would yield greater net benefit than a changing management for all patients or no patients (Figure 4).

## 4 Discussion

HL refers to an individual's ability to access, understand, and utilize health information and services to make informed decisions and manage their own health effectively [1]. The goal of this study was to create a practical method to screen patients for limited HL, without creating an additional burden for patients or healthcare professionals. Our findings show that ML algorithms can predict which patients exhibit limited HL with data that is readily available in patients' charts or can be easily obtained during a patient's visit. However, our algorithm is far from finalized and should serve more as a proof of concept instead of a clinically useful model.

A study utilizing natural language processing and ML to classify HL from secure communication between patients and healthcare providers demonstrated that these algorithms can as well, with modest accuracy, predict limited HL [37]. However, their suggested method is complicated to incorporate into the clinical workflow since it relies heavily on the use of secured messaging systems for patient–provider interactions, which are not always available. The variables used to develop algorithms in this study are already available or easy to obtain, which makes it a more feasible method to apply in practice.

Accurately identifying patients with limited HL would enable healthcare professionals to tailor care to a patient's specific needs instead of a one‐size‐fits‐all approach. Interventions targeting HL can lead to changes in health behavior and thereby improve outcomes for populations most at risk for health inequality [12]. A study by Kee et al. demonstrated that tailoring preoperative education and postoperative instructions to a patient's level of HL can significantly impact postoperative outcomes [38]. Furthermore, limited HL leads to avoidable waste of medical resources. A study in patients with degenerative lumbar disease found that patients with limited HL were more likely to overutilize more expensive drugs and select invasive interventions before exhausting less costly and more conservative measures [4].

In an ideal situation, an externally validated algorithm tested on a substantially larger population could serve as a great tool to predict limited HL. However, this is not yet the case. As of now, gathering the required information to run the algorithm would be more time consuming than just administering the NVS in the first place. Further development of algorithms may, however, result in clinically useful tools in the future. This study reinforces the notion that creating such tools could be feasible. These tools would ensure clinicians do not need to administer lengthy and time‐consuming questionnaires, which are prone to error when administered by untrained professionals [13, 14]. Therefore, future studies should focus on increasing the sample size on which the algorithm is trained and should ideally include internal and external validation to ensure their findings are generalizable in other populations as well.

### 4.1 Limitations

There are limitations to this study. One notable limitation of this study is the absence of external validation for the developed model. While the internal validation procedures employed within the study provide insights into the performance and generalizability of the model within the data set used for training and testing, the lack of external validation introduces an element of uncertainty regarding the model's applicability to other populations.

Second, all patients were seen at a single institution. The demographics of this study's population with predominantly White and well‐educated patients with above‐average household income may not be representative of other areas, which may lead to challenges with the generalizability and usefulness of this algorithm in areas with a different population.

Third, the ML models used in this study were optimized for predicting limited HL accurately, but not for explaining the independent effect of individual factors. The model parameters cannot be employed for explanatory purposes of the independent effect of the variables on HL. However, preceding work by our study group employed logistic regression on a part of the data used for this study for explanatory purposes [2].

Another limitation of this study is that we did not perform a formal sample size calculation, which is typically not standard practice in ML studies of this nature. Our study included 753 patients for model selection, which provides a solid foundation for a proof‐of‐concept investigation. However, it's important to note that this study was not designed to validate the model for immediate clinical implementation.

Furthermore, this study uses data from the ADI and SVI, which are sociodemographic indices specific to the United States, and as such, the models developed do not seamlessly translate to healthcare systems and populations in other countries. The unique contextual factors influencing HL in diverse global settings may not be adequately captured by the US‐centric indices utilized in this study.

As a tertiary spine centre, selection bias may arise. However, we believe this bias was effectively mitigated by adhering to the inclusion and exclusion criteria and selecting patients at random. Additionally, the inclusion of patients with both surgical and nonsurgical interventions increases the generalizability of our findings to other patient populations. Lastly, response fatigue should be considered. Although the average duration for completion of the survey was not measured, the survey took less 10 min to complete in all patients. Therefore, we do not believe response fatigue influenced our results.

## 5 Conclusion

Our study shows that Elastic‐Net Penalized Logistic Regression had the best performance when compared with other ML algorithms with a c‐statistic of 0.766, calibration slope of 1.044, calibration intercept of −0.037, and a Brier score of 0.179. While this algorithm is far from readily being used in clinical practice for predicting limited HL, we believe it serves as a decent proof‐of‐concept with its reasonable accuracy and good calibration. Future studies should externally validate whether this algorithm is widely applicable to a more diverse US population and if it can be applied outside the orthopaedic spine population. Ideally, a more extensively tested an externally validated algorithm with a larger sample size would help healthcare providers identify patients that may benefit from additional time and/or educational resources to prevent adverse outcomes in patients with limited HL, without taxing the clinical workflow.

## Disclosure

Each author certifies that he or she has no commercial associations (e.g. consultancies, stock ownership, equity interest, patent/licensing arrangements, etc.) that might pose a conflict of interest in connection with the submitted article. Investigation performed at Massachusetts General Hospital, Boston USA.

## Ethics Statement

This study was approved by our institutional review board.

## Conflicts of Interest

The authors declare no conflicts of interest.

## Supporting information

Supporting information.

## Data Availability

The data that support the findings of this study are available on request from the corresponding author. The data are not publicly available due to privacy or ethical restrictions.
