# Supplementary material for: Development of Machine Learning Algorithms for Identifying Patients With Limited Health Literacy
Source: J Eval Clin Pract. 2024 Nov 22;31(1):e14248. doi: 10.1111/jep.14248 (PMC11582738; doi:10.1111/jep.14248)
Supplement: Supplementary file 1 — Supporting information. [file JEP-31-0-s001.docx]

**Appendix I.** Newest Vital Sign nutritional label and accompanying questions.
